# Socio-economic inequality and inequity in use of health care services in Kenya: evidence from the fourth Kenya household health expenditure and utilization survey

**DOI:** 10.1186/s12939-019-1106-z

**Published:** 2019-12-18

**Authors:** Stefania Ilinca, Laura Di Giorgio, Paola Salari, Jane Chuma

**Affiliations:** 1 Global Brain Health Institute, Trinity College Dublin, Dublin, Ireland; 2 European Centre for Social Welfare Policy and Research, Vienna, Austria; 3 The World Bank, Washington, DC USA; 4 Swiss Tropical and Public Health Institute, Basel, Switzerland; 5 Institute of Pharmaceutical Medicine (ECPM), University of Basel, Basel, Switzerland

**Keywords:** Inequality, Inequity, Kenya, Health care, Outpatient care, Inpatient care, Prevention and promotive care, Socio-economic determinants

## Abstract

**Background:**

Kenya is experiencing persistently high levels of inequity in health and access to care services. In 2018, decades of sustained policy efforts to promote equitable, affordable and quality health services have culminated in the launch of a universal health coverage scheme, initially piloted in four Kenyan counties and planned for national rollout by 2022. Our study aims to contribute to monitoring and evaluation efforts alongside policy implementation, by establishing a detailed, baseline assessment of socio-economic inequality and inequity in health care utilization in Kenya shortly before the policy launch.

**Methods:**

We use concentration curves and corrected concentration indexes to measure socio-economic inequality in care use and the horizontal inequity index as a measure of inequity in care utilization for three types of care services: outpatient care, inpatient care and preventive and promotive care. Further insights into the individual and household level characteristics that determine observed inequality are derived through decomposition analysis.

**Results:**

We find significant inequality and inequity in the use of all types of care services favouring richer population groups, with particularly pronounced levels for preventive and inpatient care services. These are driven primarily by differences in living standards and educational achievement, while the region of residence is a key driver for inequality in preventive care use only. Pro-rich inequalities are particularly pronounced for care provided in privately owned facilities, while public providers serve a much larger share of individuals from lower socio-economic groups.

**Conclusions:**

Through its focus on increasing affordability of care for all Kenyans, the newly launched universal health coverage scheme represents a crucial step towards reducing disparities in health care utilization. However in order to achieve equity in health and access to care such efforts must be paralleled by multi-sectoral approaches to address all key drivers of inequity: persistent poverty, disparities in living standards and educational achievement, as well as regional differences in availability and accessibility of care.

## Background

The achievement of equity in health and access to health care have been key policy priorities for the Kenyan Government since the country achieved independence. Progress accelerated after 1994 with the development of the Health Policy Framework and the National Health Sector Strategic Plans and with the recognition of health sector improvement as a critical priority in the Vision 2030 long-term development plan. In resonance with the Constitution of Kenya (2010) and the aim to realize fundamental human rights for the entire population, the Kenya Health Policy 2012–2030 set as its core goal the creation of “equitable, affordable and quality health and related services at the highest attainable standards for all Kenyans” [1]. A key priority in this context is to address persistent socio-economic inequities in access to care by ensuring health services are available to all those who need them and that no undue and prohibitive financial burden is placed on those seeking care [2, 3].

Despite considerable health gains over the last decades, marked geographical and socio-economic inequalities in health have persisted in Kenya [4–7], reflecting a widespread situation in sub-Saharan African countries [8–10]. Not only do poorer individuals have worse health than wealthier population groups, but they are less able to access needed care services. While comparative studies tracking inequalities in general outpatient and inpatient care use in the region are scarce, there is convincing evidence of inequalities in use of maternal and child health care service [10–13], access to HIV-specific care and treatment [14] and preventive care [15]. Furthermore, evidence from country specific studies converges on the conclusion, that impoverished or lower socio-economic status individuals face larger barriers to accessing needed care services [16–18].

Within this regional context, socio-economic inequalities in care utilization in Kenya are well documented for a host of care services including reproductive, maternal and child care [19, 20], preventive care and immunization [20, 21], urgent care [22], inpatient and outpatient care utilization [23, 24]. Poverty levels are strongly associated with demand and availability of high quality, formal health care services [25]. Individuals from poorer households show lower propensity to seek care in health facilities (as opposed to relying on traditional healers or self-treating with medicines bought directly from pharmacies) when facing health problems and illness [26] and the quality of service providers is lower in poorer areas [27].

Further socio-economic inequalities in care use can be observed between health care sectors in Kenya. The private sector is more heterogeneous in terms of types of care providers and primarily serves wealthier individuals, whereas those from poorer households more commonly rely on public care providers or use lower standard, often unlicensed, private care facilities [28, 29]. These differences can be traced back to spatial factors (e.g. distance to any health care provider and to high quality health care facilities) and to variations in care costs and out-of-pocket payments between public and private care providers [30, 31]. While services in private clinics and in private and public hospitals are still subject to considerable user fees, care is free in public health centres and dispensaries (i.e. level 2 and level 3 facilities). Issues with care accessibility are exacerbated by low coverage of health insurance across Kenya (under 20% in 2018, although following a constant growth from only 10% in 2007) and its unequal distribution, disproportionately favouring individuals who are wealthier, formally employed and have higher educational achievement [24, 32].

As attempts to address the high levels of inequality in access to care through the establishment of contributory and voluntary insurance schemes have yielded limited results [33], the Kenyan government has launched a concerted effort to ensure all Kenyans and particularly those from disadvantaged groups and regions can access needed care through a national, universal health coverage scheme [34].

In 2017, universal health coverage (UHC) was identified as one of the four pillars of the Kenyan plan for socio-economic growth, with the aspirations of achieving full population coverage, subsidizing all costs for essential health services and cutting medical out-of-pocket expenses for Kenyan households in half by 2022. Following on this pledge, in December 2018 the Kenyan government launched the first phase of an implementation strategy for UHC. Four counties with different population health profiles and facing diverse challenges were included in the pilot phase (expected to reach over 3 million individuals): Kisumu (high rates of communicable diseases), Nyeri (high rates of non-communicable diseases), Machakos (high incidence of road traffic accidents) and Isiolo (one of the poorest Kenyan counties with high rates of maternal mortality). The pilot consists in removing user fees at level 4 and 5 hospitals while compensating facilities for the revenue lost and strengthening availability of pharmaceutical and non-pharmaceutical supplies at all levels of care. Based on the lessons learned from this pilot, the government plans a national rollout of UHC to all 47 counties by 2022.

As Kenya embarks on this ambitious policy project, monitoring and evaluation efforts are crucial to assess policy impact. Namely, it is important to establish whether the rollout of UHC will lead to the expected gains in equity in health and access to care and social inclusion, among other objectives. In this context, our study draws on nationally representative data collected shortly before the launch of the UHC pilot to establish a baseline assessment of socio-economic inequality and inequity in health care utilization in Kenya. By setting a basis for comparison, both at national and at county level, our estimates provide an opportunity to track progress towards eliminating inequality and inequity in care use and improving accessibility and availability of care alongside the gradual expansion of UHC throughout the country. To account for structural differences between care sectors, variations in accessibility and organization of diverse care services and the potential of differential policy impact, we independently estimate levels of inequality and inequity in care use by care type (i.e. outpatient, preventive/promotive and inpatient services) and by provider ownership (i.e. private, faith-based and public facilities).

To the best of our knowledge, our study contributes the most comprehensive analysis of inequality in health services use in Kenya to date, by accounting for spatial differences (county level analysis), sector differences (provider ownership) and type of care services provided. Whereas previous studies have focused on specific types of care services and care providers or relied on local samples, we use nationally representative data to generate national and county level estimates across care and provider types. Furthermore, to understand to what extent socio-economic inequality in care use (i.e. different levels of care utilization between poorer and richer individuals) is driven by differences in need for care rather than by ability to access needed care, we also run analyses of inequity, which expand the current knowledge base. Through decomposition analysis, we separate variation determined by differences in care needs from inequality that can be traced back to demographic, socio-economic and regional characteristics of individuals and households. In their entirety, our results provide a detailed overview of socio-economic inequality and inequity in care use in Kenya and of the individual and household level factors that drive these dynamics, therefore providing crucial insights for policy intervention.

## Data and methods

### Data and measurement

All analyses presented in this study are based on micro-data collected as part of the 2018 Kenya Household Health Expenditure and Utilization Survey (KHHEUS) in April and May 2018 in all 47 Kenyan counties. KHHEUS explores health spending, utilization of health services and health insurance coverage for a representative sample of households at national and county level using a computer assisted personal interview technique [24]. We have maintained for analysis data on all individuals who provided valid responses for health care utilization and household expenditure. The final analysis sample consists of 141,035 individuals from 31,636 households.

We consider three main health care utilization variables: outpatient care use (binary variable indicating whether the respondent has used outpatient care in the previous 4 weeks), preventive care use (binary variable for preventive care use during the last 4 weeks) and inpatient care use (binary variable indicating whether the respondent has been admitted to hospital during the past 12 months). Outpatient care was further categorized as public (if the care provider was reported as national referrals, county government hospital, government-based health centre, government dispensary or village health worker), private (private hospital, private clinic, nursing/maternity home, laboratory/diagnostic centre, chemist/pharmacy/shop) or faith-based/non-profit (faith-based hospital, faith-based health centre, faith-based dispensary, NGO clinic). Similarly, inpatient care was coded as public (national referrals, county government hospital, government health centre), private (if provided in private hospital, private health centre, nursing/maternity home) or faith-based/non-profit (faith-based hospital, faith-based health centre).

We used total household expenditure (a proxy for household income), calculated as the sum of household food and non-food expenditure and consumption, as the ranking variable in all analyses. Values are equivalized using the Anzagi-Bernard scale, assigning a weight of 1 to all household members aged 15 and older, a 0.65 weight to children aged 5 to 14 and a 0.24 weight for all children younger than 4.

Poor self-reported health (binary indicators of less than good health), the presence of chronic conditions (including hypertension, other cardiac disorders, diabetes, asthma, tuberculosis, other respiratory disorders, HIV/AIDS, cancer) and the presence of medical conditions are used as indicators of individual health status. Educational achievement (primary, secondary and tertiary education with ‘less than primary education’ as reference category) and employment status (no employment, informal employment with ‘formal employment’ as reference category) are defined both for each respondent and for the household head. Finally, we consider a set of household characteristics reflecting living standards, including the size of the household, urban residence and the availability of finished floors, finished walls, electricity, piped water and a flush toilet.

### Analytical approach

Following a well-established literature in the field, we use concentration curves and concentration indexes to measure socio-economic inequality in care use and the horizontal inequity index as a measure of inequity in care utilization [35–38].

Throughout the analysis, we subscribe to an understanding of inequality as the condition of being different or unequal. This pertains to any observed variation in health status or dissimilarities in the utilization of health resources between different groups [39]. We use the term inequity when referring to those inequalities that can be considered unfair or unjust, and that are unnecessary and avoidable [39, 40]. In the following, we deem as fair and necessary all those differences in care utilization between groups that are determined by differences in health status, while all remaining inequality after accounting for variation in care needs is considered inequitable. This interpretation is grounded in the “right of every human being to the enjoyment of the highest attainable standard of physical and mental health, without distinction as to race, religion, political belief, economic or social condition” [41]. and the same approach is reflected in universal health coverage policy, which aims to ensure all people have access to needed care, without discrimination and without being exposed to undue financial hardship.

Starting from a definition of equality as the state where each individual uses the same amount of care resources as all other members of a population, irrespective of their socio-economic and health status, we use concentration curves to establish departures from this baseline. Concentration curves plot the cumulative percentage of a given health utilization variable against the cumulative population proportion, ranked by socioeconomic status (running from the poorest to the richest population groups). Inequality increases as concentration curves diverge from the equality line. A simple and synthetic measure of total inequality in care use is offered by the concentration index (CI), which measures the area between the concentration curve and the line of equality. The CI varies in the (− 1, 1) interval and takes negative values when the care use variable is disproportionately concentrated among the poor, whereas a positive value indicates that inequality favours the wealthier. As the CI approaches zero, lower levels of inequality are present.

We calculate concentration indices for the probability to use each of three types of care services: outpatient care, inpatient care and preventive and promotive care (including family planning, immunization, voluntary counselling and testing, ante/post-natal care). As the care utilization variables are binary, we apply the scale correction proposed by Erreygers [42, 43] and resulting in the corrected concentration index (CCI).

Significant differences in inequality levels between different types of care are assessed via dominance tests for concentration curves [38].

We then carry out a decomposition analysis to provide further insights into the individual and household characteristics that determine observed inequality. Building on a regression analysis technique for the decomposition of the CI [44], we apply an extension for non-linear models based on a partial effects representation [38, 45]. In this specification, the CI is expressed as the sum of the contributions of all considered factors (obtained from the elasticity of care use with respect to each factor and the concentration index of each factor) and an error component (called the generalized concentration index for the error term). This approach allows us to decompose the value of the concentration index into the contributions of several key factors: household expenditure, care needs (health status), age and gender, household composition, educational achievement, employment and region of residence.

Estimates of inequity in care use through the horizontal inequity (HI) index embody the principle that individuals with equal needs for care should have equal care utilization. We use the indirect standardization method to derive HI values by calculating differences between actual and need-predicted care utilization [46, 47]. By separating the determinants of care use into a group describing care needs and a group reflecting other socio-economic characteristics (non-need), we can use a logistic regression model to estimate how much care each individual would receive, if they were treated equally to other individuals in the sample with equal care needs (i.e. need-predicted care utilization). Inequity in care use (HI) can then be calculated as the difference between need-predicted care use and actual (observed) care use. All data analyses were carried out in Stata 15 [48].

## Results

Descriptive statistics for dependent and independent variables by total household expenditure quintiles are presented in Table 1. Levels of care utilization disaggregated at county level are available in Additional file 1.

**Table 1:** Descriptive statistics by total household expenditure quintile (in %, unless otherwise specified)

	Poorest quintile	2nd quintile	3rd quintile	4th quintile	Richest quintile
Female	50.67	50.99	50.53	50.76	50.28
Age (mean, in years)	26.63	25.25	24.96	25.47	27.48
0–14 years	21.97	21.76	20.75	19.60	15.92
15–34 years	18.91	18.76	19.66	20.28	22.39
35–54 years	16.90	18.45	19.24	20.57	24.84
55+ years	21.10	19.71	19.46	19.60	20.13
Health status					
Poor self-reported health	13.84	11.64	11.07	10.85	10.35
Has chronic condition	8.70	9.18	9.38	10.04	11.12
Has medical condition	2.34	2.40	2.70	2.77	2.57
Household characteristics					
Household size (mean, persons)	6.8	6.5	6.1	5.5	4.4
Finished floor	14.05	23.99	37.40	52.30	76.66
Finished wall	16.08	25.00	35.48	47.11	67.08
Electricity	10.01	17.41	27.18	42.62	67.67
Piped water	26.27	34.27	40.08	47.72	58.94
Flush toilet	1.98	3.63	5.28	11.59	27.68
Urban residence	17.17	21.69	29.11	37.93	56.26
Individual employment					
Formal employment	1.03	1.60	2.54	4.38	11.11
Informal employment	19.04	19.49	21.45	23.16	26.95
Not employed	79.93	78.90	76.01	72.46	61.94
Household head employment					
Formal employment	3.67	5.55	8.19	12.73	24.79
Informal employment	55.79	58.66	58.87	57.96	52.13
Not employed	40.54	35.79	32.93	29.31	23.09
Individual education level					
Less than Primary	25.03	21.16	20.84	20.89	16.56
Primary	59.32	59.00	54.68	49.40	39.34
Secondary	14.09	17.28	20.67	22.91	27.21
Tertiary	1.57	2.56	3.81	6.80	16.88
Household head education					
Less than Primary	35.12	27.06	24.90	22.77	13.47
Primary	49.12	51.22	46.65	40.67	28.39
Secondary	14.31	18.37	22.69	26.27	32.34
Tertiary	1.45	3.35	5.77	10.29	25.79
Care utilization					
Outpatient care	12.10	12.43	13.65	14.09	15.95
Private	3.09	3.29	4.22	5.65	8.85
Public	10.10	10.63	11.14	10.63	9.99
Faith-based	0.72	0.71	0.87	1.01	1.29
Inpatient care	2.61	2.83	2.92	3.34	4.37
Private	0.61	0.67	0.85	1.03	1.77
Public	1.68	1.73	1.65	1.91	2.05
Faith-based	0.26	0.40	0.41	0.42	0.58
Preventive & promotive care	3.35	3.61	4.02	4.67	6.09

Richer individuals in Kenya report better health status than those in poorer households, although the prevalence of chronic and medical conditions is higher in richer population groups, as is the average age. Higher educational achievement (particularly at tertiary level) and formal employment (both at individual and household head level) are exceedingly more likely in higher expenditure quintiles, and extremely low among the poorest groups. Richer households tend to be smaller (4.4 household members in the top expenditure quintile as compared to 6.8 in the lowest), located in urban settings and report considerably higher availability levels of modern amenities.

Health care utilization in Kenya increases with socio-economic status. Larger shares of rich individuals (5th expenditure quintile) as compared to poorer population groups use outpatient care, inpatient care and preventive care. A particularly large gap is observed for preventive care and hospital admissions, from 3.3 to 6.1% and respectively, from 2.6 to 4.3% in richer groups. Care utilization increases with household expenditure quintile irrespective of the type of provider considered, with the noteworthy exception of outpatient care provided in public facilities, the share of which remains stable.

### Inequality and inequity in care use

Our results show significant pro-rich inequality in the use of all types of care services in Kenya (Table 2). Concentration indexes for outpatient, inpatient care and preventive and promotive care are all positive and statistically significant, indicating richer individuals use disproportionately more health services than their respective population proportion, while lower socio-economic status groups access a significantly lower share of care resources, irrespective of service type.

**Table 2:** Inequality and inequity in care utilization by care type

Care type	Concentration index	Std. Err	*p*-value	Horizontal inequity index	Std. Err	*p*-value
Outpatient	0.0212***	(0.0040)	*0.0000*	0.0096*	(0.0040)	*0.0164*
Preventive & promotive	0.0256***	(0.0025)	*0.0000*	0.0156***	(0.0025)	*0.0000*
Inpatient	0.0149***	(0.0021)	*0.0000*	0.0048*	(0.0020)	*0.0223*

Note: Standard errors reported in parentheses. * *p* < 0.05, *** *p* < 0.001

The pro-rich distribution is maintained even after controlling for different care needs between socio-economic groups, with values for HI indexes positive and significant for all care types. The estimates are overall robust to changing the socio-economic status indicator used as a ranking variable from total to non-food household expenditure (see Additional file 2).

**Fig. 1:**
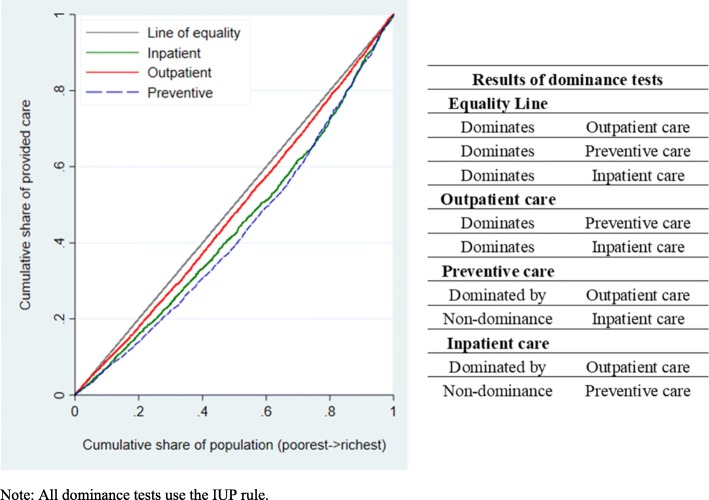
Joint plot of concentration curves for outpatient, preventive and inpatient care utilization. *Note: All dominance tests use the IUP rule*

We use tests of dominance between concentration curves to establish whether one type of care is more unequally distributed than another. To do so we investigate whether one curve lies significantly above another at all points in the graph (dominates), overlaps the other at some point across the distribution (non-dominance) or lies significantly below another (is dominated). In line with the results for concentration indexes by care type, we find dominance of the equality line over the concentration curves for all types of care utilization (i.e. the equality line lies significantly above the concentration curves) (Fig. 1). Both preventive and promotive health services and inpatient services are more unequally distributed than outpatient care services (i.e. outpatient care concertation curves dominate), but we find no evidence of a marked difference in inequality in care use between the former two (non-dominance between concentration curves for preventive and inpatient care utilization).

### Decomposition of inequality in care use

**Fig. 2:**
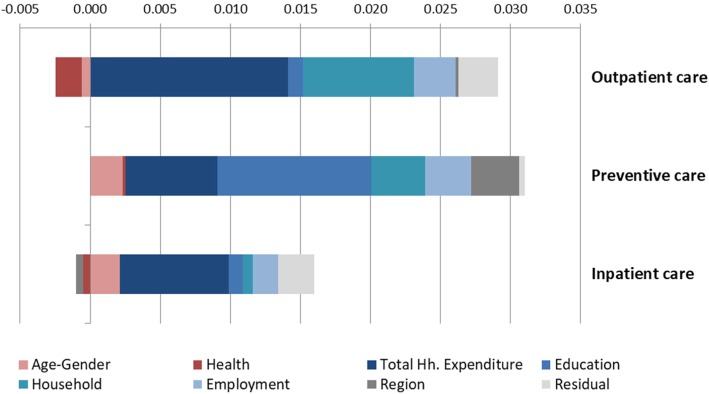
Decomposition of concentration indexes (by care type)

Figure 2 presents the decomposition of the CI into the contributions of several key factors. The distribution of the bars spanning from the origin point indicate that contributions of different categories of factors can pull inequality either towards richer individuals (positive values, right-hand side) or poorer individuals (negative values, left-hand side). The size of the bars relative to each other indicate the overall contribution of each factor to total inequality, therefore longer bars represent larger contributions to the total CI.

The magnitude of the contribution of each factor depends on: (i) how sensitive health care use is to variation in the given factor - i.e., its elasticity with respect to it; and (ii) how equal the distribution of a given factor is with respect to the socio-economic status of a household - i.e., its concentration index. Consequently, the largest contributions to overall inequality are relative to those factors that are both unequally distributed and strongly associated with health care use.

The main driving factors of socio-economic inequality in health care use in Kenya are total household expenditure, educational achievement, household characteristics and living standards, all disproportionately distributed in favour of richer individuals and better off households. The educational achievement of the individuals using health services and of the household head are particularly relevant for the distribution of preventive health service utilization and relatively less relevant for inpatient and outpatient care, whereas household characteristics explain a large portion of inequality in outpatient care utilization.

Health care needs drivers (measured here as self-reported health status, number of chronic conditions and the presence of medical conditions) explain a very low share of total socio-economic inequality in health service use. It is also interesting to note that in the case of preventive care and inpatient care, age and gender contribute positive values to the CI and drive pro-rich inequality. This result is explained by a higher concentration of young and adult household members in richer households, while poorer households tend to have more young children or older adults in their composition. Poorer health status is generally concentrated among lower socio-economic groups, with the noteworthy exception of chronic conditions.

The region of residence explains a considerable part of total inequality in preventive care utilization but only minor shares of inequality in outpatient and inpatient care use, suggesting that spatial inequalities are not equally pronounced and relevant for all care types.

### Local variation in inequality in care use

We find pronounced differences in levels of inequality in health care utilization between Kenyan counties, for all types of care services analysed (Table 3). Wherever statistically significant, inequality favours richer individuals. Preventive care services are most frequently unequally distributed (in half of Kenyan counties), with particularly high concentrations in the Rift Valley and Western regions. Only five of the 47 counties register significant levels of pro-rich inequality in all three types of care services considered.

**Table 3:** Inequality in care utilization at the county level (by care type)

	No. of observations	GCP per capita^a^	Outpatient		Preventive		Inpatient	
County			CI	*Std.err*	CI	*Std.err*	CI	*Std.err*
Mombasa	2025	271,039	−0.0044	*0.0241*	0.0213	*0.0145*	0.0209*	*0.0102*
Kwale	3536	101,725	−0.0060	*0.0234*	0.0065	*0.0115*	0.0054	*0.0096*
Kilifi	3585	82,405	0.0390	*0.0211*	0.0261**	*0.0094*	0.0198*	*0.0083*
Tana River	3488	106,894	−0.0154	*0.0268*	−0.0070	*0.0078*	0.0037	*0.0061*
Lamu	2792	244,379	0.0638**	*0.0203*	0.0150	*0.0082*	0.0007	*0.0089*
Taita/Taveta	2302	139,053	0.0076	*0.0272*	0.0149	*0.0154*	0.0038	*0.0087*
Garissa	3119	89,502	0.0634***	*0.0171*	0.0117	*0.0066*	0.0198**	*0.0067*
Wajir	4112	79,468	0.0169	*0.0101*	0.0284***	*0.0073*	−0.0037	*0.0059*
Mandera	4456	48,442	0.0678***	*0.0173*	0.0392**	*0.0127*	0.0314*	*0.0123*
Marsabit	3210	106,734	0.0414*	*0.0181*	0.0248***	*0.0073*	0.0352***	*0.0098*
Isiolo ^b^	3161	100,904	0.0285*	*0.0125*	0.0438***	*0.0123*	0.0215**	*0.0082*
Meru	2884	154,537	0.0237	*0.0219*	0.0348	*0.0131*	0.0154	*0.0101*
Tharaka-Nithi	2602	169,141	−0.0450	*0.0231*	0.0249	*0.0150*	0.0013	*0.0099*
Embu	2431	183,418	0.0199	*0.0269*	0.0334**	*0.0123*	−0.0046	*0.0110*
Kitui	2728	91,580	0.0685**	*0.0230*	0.0122	*0.0105*	0.0064	*0.0079*
Machakos ^b^	2975	193,460	0.0219	*0.0219*	0.0150	*0.0107*	0.0079	*0.0099*
Makueni	3031	104,161	0.0450*	*0.0185*	0.0073	*0.0109*	0.0118	*0.0063*
Nyandarua	2636	350,321	0.0508*	*0.0227*	0.0271*	*0.0131*	0.0028	*0.0087*
Nyeri ^b^	2390	214,885	0.0921***	*0.0231*	0.0081	*0.0140*	0.0092	*0.0106*
Kirinyaga	2128	162,666	0.0838***	*0.0240*	0.0149	*0.0112*	0.0012	*0.0122*
Murang’a	2417	156,392	0.0335	*0.0232*	0.0019	*0.0107*	0.0085	*0.0078*
Kiambu	2340	221,467	0.0253	*0.0197*	0.0400	*0.0125*	0.0209*	*0.0095*
Turkana	3578	69,775	0.0563*	*0.0274*	0.0740***	*0.0200*	0.0222*	*0.0107*
West Pokot	4097	69,589	0.0045	*0.0143*	0.0136	*0.0070*	0.0060	*0.0052*
Samburu	2888	90,143	0.0586**	*0.0198*	0.0288**	*0.0092*	0.0104	*0.0073*
Trans Nzoia	3218	108,607	0.0447**	*0.0146*	0.0192**	*0.0074*	0.0180*	*0.0083*
Uasin Gishu	3222	138,350	−0.0054	*0.0155*	0.0315**	*0.0110*	0.0084	*0.0071*
Elgeyo	3271	328,575	0.0088	*0.0142*	0.0024	*0.0093*	−0.0003	*0.0075*
Nandi	3224	121,149	0.0135	*0.0168*	0.0067	*0.0095*	0.0031	*0.0072*
Baringo	2607	127,437	−0.0025	*0.0210*	0.0055	*0.0090*	−0.0103	*0.0125*
Laikipia	2704	154,840	−0.0058	*0.0235*	0.0270**	*0.0101*	0.0089	*0.0083*
Nakuru	2698	245,999	0.0261	*0.0156*	0.0164*	*0.0081*	0.0116	*0.0063*
Narok	2921	160,580	0.0346	*0.0222*	0.0387***	*0.0098*	0.0394***	*0.0098*
Kajiado	2325	119,557	0.0513	*0.0182*	0.0387***	*0.0115*	0.0219**	*0.0084*
Kericho	2835	141,047	0.0252	*0.0198*	0.0771***	*0.0144*	0.0283**	*0.0096*
Bomet	3428	167,777	0.0492**	*0.0159*	0.0283***	*0.0073*	0.0062	*0.0057*
Kakamega	3470	95,667	−0.0101	*0.0210*	0.0159*	*0.0074*	0.0267**	*0.0088*
Vihiga	3192	92,572	−0.0014	*0.0237*	0.0312*	*0.0127*	0.0159	*0.0104*
Bungoma	3663	97,986	0.0328*	*0.0160*	0.0200*	*0.0082*	0.0084	*0.0081*
Busia	3575	154,722	0.0046	*0.0220*	0.0421***	*0.0111*	0.0213	*0.0112*
Siaya	2812	94,714	0.0426	*0.0168*	0.0330***	*0.0118*	0.0001	*0.0116*
Kisumu ^b^	2699	168,095	0.0149	*0.0262*	−0.0029	*0.0149*	0.0287	*0.0177*
Migori	3501	87,960	−0.0161	*0.0207*	−0.0197	*0.0119*	0.0192*	*0.0095*
Homa Bay	3176	99,227	0.0206	*0.0192*	0.0037	*0.0077*	0.0447***	*0.0121*
Kisii	2886	118,858	0.0321*	*0.0141*	0.0136	*0.0092*	0.0185*	*0.0082*
Nyamira	2796	144,512	0.0355*	*0.0152*	0.0135	*0.0124*	0.0113	*0.0087*
Nairobi City	1998	317,700	−0.0001	*0.0241*	0.0136	*0.0145*	0.0213	*0.0110*

Note: * *p* < 0.05, ** *p* < 0.01, *** *p* < 0.001

^a^Gross county product (GCP) per capita at current prices (Ksh), in 2017 - Kenya Bureau of Statistics [49]

^b^County included in the universal health coverage pilot

Out of the four counties included in the UHC pilot, we find systematic inequality in all three types of care use only in Isiolo. We find no statistically significant inequality in care use in Kisumu and Machakos, while in Nyeri only outpatient care is significantly distributed in favour of richer population groups.

### Inequality in private health service utilization

The private health sector in Kenya plays a key role in national health care provision, serving an important share of the population across socio-economic status groups. However, previous analyses have found inequalities in access to care provided in privately owned facilities. Our analysis confirms these results, revealing marked pro-rich inequality and inequity in private care utilization. Richer Kenyans use privately owned care facilities more frequently than lower socio-economic status individuals do, even after controlling for differences in care needs. Pro-rich inequality in private care use is large and statistically significant in the case of both outpatient and inpatient care services (Table 4). We also find pro-rich inequality in the use of care services provided in non-profit or faith-based facilities, irrespective of whether services were offered on an outpatient or inpatient basis.

**Table 4:** Concentration indexes for outpatient and inpatient care utilization (by provider ownership) – full sample estimates

Outpatient care			Inpatient care			
Care type	Concentration index	Std.err.	*p-value*	Concentration index	*Std.err.*	*p-value*
Private	0.0542***	(0.0032)	*0.0000*	0.0113***	(0.0014)	*0.0000*
Non-profit/faith-based	0.0047***	(0.0011)	*0.0001*	0.0025**	(0.0008)	*0.0015*
Public	−0.0135***	(0.0033)	*0.0000*	0.0023	(0.0014)	*0.1175*

Note: Standard errors reported in parentheses. ** *p* < 0.01, *** *p* < 0.001

Conversely, lower socio-economic status individuals disproportionately use outpatient services offered in publicly owned facilities (negative and significant CI). We find no statistically significant inequality in the utilization of public inpatient care in Kenya.

To separate the effect of socio-economic status on the probability to use health services from its association with the type of care provider an individual in need of care will use, we repeated the same analysis on the sub-sample of care users (Table 5). Measured inequality increases considerably, both in the case of outpatient and inpatient care use, favouring richer individuals for privately owned services. Conversely, we find significant pro-poor inequality in the use of health services offered by publicly owned facilities.

**Table 5:** Concentration indexes for outpatient and inpatient care utilization (by provider ownership) – sub-sample of care users only

Outpatient care			Inpatient care			
Care type	Concentration index	Std.err.	*p-value*	Concentration index	Std.err.	*p-value*
Private	0.2405***	(0.0137)	*0.0000*	0.1948***	(0.0299)	*0.0000*
Non-profit /faith-based	0.0133	(0.0068)	*0.0527*	0.0137	(0.0202)	*0.4982*
Public	−0.2387***	(0.0137)	*0.0000*	−0.1751***	(0.0310)	*0.0000*

Note: Standard errors reported in parentheses. *** *p* < 0.001

In other words, if we consider just the population who seeks care, lower socio-economic status individuals will rely on services provided by publicly owned facilities and non-profits more frequently, while richer individuals will represent the largest proportion of care users of for profit, privately owned facilities. Plots of concertation curves by care type and provider ownership for the full sample and for care users only are provided in Additional file 3.

## Discussion

Our study provides an overview of socio-economic inequality and inequity in health care use in Kenya and can act as a baseline assessment for evaluating the impact of the newly implemented universal health coverage policy. The results confirm the persistence of marked pro-rich inequality in the utilization of all health care services analysed. This suggests that the series of health reforms implemented over the last decades have not led to the achievement of an equitable and accessible health care system for all Kenyans and that further, coordinated policy intervention is needed.

The remaining gap in accessibility and affordability of services affects all types of care, but it is particularly pronounced for preventive and promotive care and inpatient care utilization. While user fees were removed for dispensaries and health centres (level 2 and level 3 facilities), care in hospitals (level 4 and level 5 facilities) was still subject to burdensome fees, which can discourage poorer individuals to seek needed care. Furthermore, a host of indirect costs (e.g. costs of transportation and loss of working time) compound the burden for individuals from poorer households [50].

Even though affordability of care remains a major concern in Kenya, large inequalities in preventive and promotive care use suggest additional barriers to care contribute to lower levels of care utilization among poorer individuals. Our decomposition analysis results emphasize that inequality in living standards and educational achievement explain the largest part of total inequality in health services utilization. As this is particularly the case for preventive care services, our findings constitute further evidence that efforts to improve affordability of care must be paralleled by concerted public health and health promotion initiatives. The results resonate with previous studies, which have found that costs of access but also lack of information and cultural barriers lead to marked differences in care-seeking behaviour by socio-economic status, with poorer individuals less likely to seek care in case of illness but also more likely to choose non–modern health care providers, such as traditional healers [26, 50–52]. Investments in reducing poverty, increasing awareness of quality of care across sectors and incentivizing the use of formal medical providers are likely to contribute significantly to the reduction of inequalities in care use.

The large contribution of household living standards to inequality in care use both for preventive and for outpatient care utilization, in conjunction with the results from the county level analysis, indicate that limited accessibility of health care facilities in the poorest communities might also affect access to care [50, 53–55]. The creation of an equitable health care system in Kenya should therefore also include a focus on improving the geographical coverage of care facilities and their balanced distribution across all counties and localities, emphasizing development of health care delivery capacity in the areas most affected by poverty.

Confirming previous results [17, 18], we found pro-rich inequality in health service use to be significantly higher for care provided in privately owned facilities, while publicly owned care providers serve a much larger share of individuals from lower socio-economic groups. In fact, outpatient care in publicly owned facilities is disproportionately used by lower income individuals, suggesting the impact of policy reforms on the elimination of user out of pocket fees at lower levels of care has contributed to the reduction of barriers in access to care. However, as a considerable share of total care services in Kenya are provided in privately owned facilities, it is important that future policies target improved accessibility and affordability of care across sectors, to avoid the exacerbation of a two-tiered health system, particularly if differences in quality of care between the two sectors persist.

We acknowledge two limitations of our study. Firstly, the results on inequity in care use presented in this paper rely on an estimate of care needs that is limited in its precision by the availability and quality of data. As the survey data we use do not include any information on symptoms, we rely on self-reported measures of chronic and medical conditions, both limited proxies for care needs. This limitation is most relevant in the case of preventive and promotive health services utilization, where the definition of care needs through indicators of health status is particularly problematic. Furthermore, we acknowledge reporting accuracy might be limited in the setting considered by our study, as individuals from lower educational and socio-economic backgrounds are likely to have lower diagnosis rates and awareness about medical conditions and chronic diseases. Secondly, we caution that estimates at county level can be affected by lower sample sizes and particularly by low rates of health service utilization in certain counties [36]. This is primarily the case for inpatient care utilization and the eastern counties of Kenya (further details on rates of care use by care type and county are provided in Additional file 3).

## Conclusions

Despite sustained policy efforts, Kenya still faces significant levels of socio-economic inequality in access to health care services, both in inpatient and in outpatient settings. What is more, richer individuals use disproportionately more care services even though they have, on average, better health status and lower care needs. Addressing these persistent inequities in care use and access to needed services is an essential step towards reducing disparities in health and wellbeing between different population groups and promoting fairer, more inclusive societies. In this context, recent efforts of the Kenyan Government to introduce and rollout universal health coverage at national level by 2022 are both timely and appropriate to ensure care services are available to all those who need them, irrespective of their socio-economic position.

Poorer individuals in Kenya often forego essential care services due to burdensome costs and even individuals from higher income households can experience significant financial hardships after seeking care for long-term conditions or severe illness. As limited access to health insurance and the considerable financial burden it places on their household are key factors limiting care utilization among Kenyans (particularly those from lower socio-economic groups) policies aimed at expanding the insurance base and increasing the affordability of care hold the greatest potential to redress the observed inequities in care use. Their success will likely hinge on the ability of Kenyan policy-makers to increase public financing for the health sector, to strengthen prepayment mechanisms and to expand the pooling of health funds both through taxation and through compulsory and voluntary contributory schemes.

While addressing affordability is a necessary condition to promote equity in health and access to care in Kenya, it will likely not be sufficient on its own. Large differences in living standards and educational achievement between income groups have a strong impact on care seeking behaviour and act as powerful drivers of inequalities in care utilization. Policies focused on care affordability must be paralleled by efforts to improve health literacy - the lynchpin to an individual’s ability to recognize care needs and address them by accessing appropriate care. A primary concern in this regard is to increase investments in strengthening primary care networks and expanding preventive and promotive care service capacity across Kenyan counties. This can ensure all Kenyans can access, understand and use health information correctly in order to maintain and improve their health status.

Finally, it is important to recognize that inequities in health and access to care spring from complex socio-economic dynamics that span beyond the scope of health policies. Multi-sectoral approaches at the local, regional and national level are necessary to address the root causes of social inequity and reduce poverty and persistent disparities between socio-economic groups. To this end, the universal health coverage policy agenda should be embedded into a larger multi-sectoral collaboration (including both private and public stakeholders) focused on addressing the determinants of health and health equity.

## Supplementary information

**Additional file 1.** Levels of care utilization in Kenyan counties (by care type).

**Additional file 2.** Inequality and inequity in care use –comparison of ranking variables (total and non-food household expenditure).

**Additional file 3.** Concentration curves for inpatient and outpatient care utilization by provider ownership.

## Data Availability

The data that support the findings of this study are available from the Ministry of Health, Government of Kenya.

## Abbreviations

CI: Concentration index
HI: Horizontal inequity index
KHHEUS: Kenya Household Health Expenditure and Utilization Survey
UHC: Universal health coverage

## References

[1] Okech TC, Lelegwe SL (2016). Analysis of universal health coverage and equity on health Care in Kenya. Global J Health Sci.

[2] Ministry of Health Government of Kenya. Kenya Health Policy 2014–2030: Towards attaining the highest standard of health. Nairobi: Ministry of Health; 2014. https://www.afidep.org/?wpfb_dl=80.

[3] Ministry of Health Government of Kenya. Transforming Health. Accelerating attainment of Universal Health Coverage. The Kenya Health Sector Strategic and Investment Plan 2013–2017. Nairobi: Ministry of Health; 2013. http://e-cavi.com/wp-content/uploads/2014/11/kenya-health-sector-strategic-investiment-plan-2013-to-2017.pdf.

[4] Achoki T, Miller-Petrie MK, Glenn SD, Kalra N, Lesego A, Gathecha GK (2019). Health disparities across the counties of Kenya and implications for policy makers, 1990-2016: a systematic analysis for the global burden of disease study 2016. Lancet Glob Health.

[5] Okiro EA (2019). Estimates of subnational health trends in Kenya. Lancet Glob Health.

[6] Were V, Buff AM, Desai M, Kariuki S, Samuels A, Ter Kuile FO (2018). Socioeconomic health inequality in malaria indicators in rural western Kenya: evidence from a household malaria survey on burden and care-seeking behaviour. Malar J.

[7] World Bank. Health Equity and Financial Protection Report – Kenya. Washington, DC: World Bank; 2012. http://documents.worldbank.org/curated/en/875011468047742115/pdf/712540WP00PUBL0Country0Report0Kenya.pdf.

[8] Pons-Duran C, Lucas A, Narayan A, Dabalen A, Menéndez C. Inequalities in sub-Saharan African women’s and girls’ health opportunities and outcomes: Evidence from the Demographic Health Surveys. J Glob Health. 2019;9(1):010410. 10.7189/jogh.09.010410 PMC6326483 30643635

[9] Umuhoza SM, Ataguba JE (2018). Inequalities in health and health risk factors in the southern African development community: evidence from world health surveys. Int J Equity Health.

[10] Yourkavitch J, Burgert-Brucker C, Assaf S, Delgado S (2018). Using geographical analysis to identify child health inequality in sub-Saharan Africa. PLoS One.

[11] Moyer CA, Mustafa A. Drivers and deterrents of facility delivery in sub-Saharan Africa: A systematic review. Reprod Health. 2013;10(1):40. 10.1186/1742-4755-10-40. 10.1186/1742-4755-10-40 PMC3751820 23962135

[12] Say L, Raine R (2007). A systematic review of inequalities in the use of maternal health care in developing countries: examining the scale of the problem and the importance of context Public health reviews. Bull World Health Organ.

[13] Wong KLM, Benova L, Campbell OMR (2017). A look back on how far to walk: systematic review and meta-analysis of physical access to skilled care for childbirth in sub-Saharan Africa. PLoS One.

[14] Sharma M, Ying R, Tarr G, Barnabas R (2015). Systematic review and meta-analysis of community and facility-based HIV testing to address linkage to care gaps in sub-Saharan Africa. Nature..

[15] Murphy A, Palafox B, O’Donnell O, Stuckler D, Perel P, AlHabib KF (2018). Inequalities in the use of secondary prevention of cardiovascular disease by socioeconomic status: evidence from the PURE observational study. Lancet Glob Health.

[16] Harris B, Goudge J, Ataguba JE, McIntyre D, Nxumalo N, Jikwana S (2011). Inequities in access to health care in South Africa. J Public Health Policy.

[17] Adedini SA, Odimegwu C, Bamiwuye O, Fadeyibi O, De Wet N (2014). Barriers to accessing health care in Nigeria: implications for child survival. Glob Health Action.

[18] Bradley E, Thompson JW, Byam P, Webster TR, Zerihun A, Alpern R (2011). Access and quality of rural healthcare: Ethiopian millennium rural initiative. Int J Qual Health Care.

[19] Keats EC, Akseer N, Bhatti Z, Macharia W, Ngugi A, Rizvi A (2018). Assessment of inequalities in coverage of essential reproductive, maternal, newborn, child, and adolescent health interventions in Kenya. JAMA Netw Open.

[20] Van Malderen C, Ogali I, Khasakhala A, Muchiri SN, Sparks C, Van Oyen H (2013). Decomposing Kenyan socio-economic inequalities in skilled birth attendance and measles immunization. Int J Equity Health.

[21] Egondi T, Oyolola M, Mutua MK, Elung’ata P (2015). Determinants of immunization inequality among urban poor children: evidence from Nairobi’s informal settlements. Int J Equity Health.

[22] O’Meara WP, Karuru S, Fazen LE, Koech J, Kizito B, Tarus C (2014). Heterogeneity in health seeking behaviour for treatment, prevention and urgent care in four districts in western Kenya. Public Health.

[23] Ministry of Health. 2013 Kenya household health expenditure and utilisation survey. Nairobi: Ministry of Health; 2014.

[24] Ministry of Health Government of Kenya (2018). 2018 Kenya household health expenditure and utilization survey. Nairobi.

[25] Musyoka PK, Korir J, Omolo J, Nzai CC (2018). An Empirical Analysis of the Effect of Poverty on Health Care Utilization in Kenya. Eur Sci J.

[26] Awiti JO (2014). Poverty and health care demand in Kenya. BMC Health Serv Res.

[27] Toda M, Opwora A, Waweru E, Noor A, Edwards T, Fegan G (2012). Analyzing the equity of public primary care provision in Kenya: variation in facility characteristics by local poverty level. Int J Equity Health.

[28] Chakraborty NM, Wanderi J, Oduor C, Montagu D (2017). Assessing provision and equity in low and middle-income country health markets: a study from Kenya.

[29] Kenya National Bureau of Statistics (2015). Kenya Demographic and Health Survey 2014. Nairobi.

[30] Subramanian S, Gakunga R, Kibachio J, Gathecha G, Edwards P, Ogola E (2018). Cost and affordability of non-communicable disease screening, diagnosis and treatment in Kenya: patient payments in the private and public sectors. PLoS One.

[31] Berendes S, Heywood P, Oliver S, Garner P (2011). Quality of private and public ambulatory health Care in low and Middle Income Countries: systematic review of comparative studies. PLoS Med.

[32] Kazungu JS, Barasa EW (2017). Examining levels, distribution and correlates of health insurance coverage in Kenya. Tropical Med Int Health.

[33] Chuma J, Okungu V (2011). Viewing the Kenyan health system through an equity lens: implications for universal coverage. Int J Equity Health.

[34] Ministry of Health G of K (2019). Universal health coverage - Everyone, everywhere. Policy Brief April.

[35] Kakwani N, Wagstaff A, van Doorslaer E (1997). Socioeconomic inequalities in health: measurement, computation, and statistical inference. J Econ.

[36] Wagstaff A, Paci P, van Doorslaer E (1991). On the measurement of inequalities in health. Soc Sci Med.

[37] Ndetei DM, Gatonga P (2011). Improving access to mental health care in Kenya. McKenzie K, editor. Ethn Inequalities Heal Soc Care.

[38] O’Donnell O, van Doorslaer E, Wagstaff A, Lindelow M (2008). Analyzing health equity using household survey data: a guide to techniques and their implementation.

[39] Kawachi I, Subramanian SV, Almeida-Filho N (2002). A glossary for health inequalities. J Epidemiol Community Health.

[40] Whitehead M, Dahlgren G (1991). What can be done about inequalities in health?. Lancet..

[41] United Nations (2013). Resolution adopted by the general assembly on 12 December 2012. 67th session.

[42] Erreygers G (2009). Correcting the concentration index. J Health Econ.

[43] Erreygers G, Van Ourti T (2011). Measuring socioeconomic inequality in health, health care and health financing by means of rank-dependent indices: a recipe for good practice. J Health Econ.

[44] Wagstaff A, van Doorslaer E, Watanabe N (2003). On decomposing the causes of health sector inequalities with an application to malnutrition inequalities in Vietnam. J Econ.

[45] van Doorslaer E, Koolman X, Jones AM (2004). Explaining income-related inequalities in doctor utilisation in Europe. Health Econ.

[46] Wagstaff A, van Doorslaer E (2000). Measuring and testing for inequity in the delivery of health care. J Hum Resour.

[47] Van de Poel E, Van Doorslaer E, O’Donnell O (2012). Measurement of inequity in health care with heterogeneous response of use to need. J Health Econ.

[48] Corp S (2017). Stata statistical software: release 15.

[49] Kenya National Bureau of Statistics (2019). Gross county product 2019. Nairobi.

[50] Kukla M, McKay N, Rheingans R, Harman J, Schumacher J, Kotloff KL (2017). The effect of costs on Kenyan households’ demand for medical care: why time and distance matter. Health Policy Plan.

[51] Mwabu G, Wang’ombe J, Nganda B (2003). The demand for medical Care in Kenya. Afr Dev Rev.

[52] Shewamene Z, Dune T, Smith CA. The use of traditional medicine in maternity care among African women in Africa and the diaspora: a systematic review. BMC Complement Altern Med. 2017;17(1):382. 10.1186/s12906-017-1886-x. 10.1186/s12906-017-1886-x PMC5541739 28768534

[53] Chuma J, Okungu V, Molyneux C. Barriers to prompt and effective malaria treatment among the poorest population in Kenya. Malar J. 2010;9(1):144. 10.1186/1475-2875-9-144. 10.1186/1475-2875-9-144 PMC2892503 20507555

[54] Burke T. F., Hines R., Ahn R., Walters M., Young D., Anderson R. E., Tom S. M., Clark R., Obita W., Nelson B. D. (2014). Emergency and urgent care capacity in a resource-limited setting: an assessment of health facilities in western Kenya. BMJ Open.

[55] Kizito J, Kayendeke M, Nabirye C, Staedke SG, Chandler CI (2012). Improving access to health care for malaria in Africa: a review of literature on what attracts patients.

