# Supplementary material for: Socio-economic inequality and inequity in use of health care services in Kenya: evidence from the fourth Kenya household health expenditure and utilization survey
Source: Int J Equity Health. 2019 Dec 18;18:196. doi: 10.1186/s12939-019-1106-z (PMC6918604; doi:10.1186/s12939-019-1106-z)
Supplement: Supplementary file 1 — Additional file 1. Levels of care utilization in Kenyan counties (by care type). [file 12939_2019_1106_MOESM1_ESM.docx]

**Additional file 1 – Levels of care utilization in Kenyan counties (by care type)**

|  | Sample size | **Inpatient care** | | **Outpatient care** | | **Preventive care** | |
| --- | --- | --- | --- | --- | --- | --- | --- |
| **County** |  | Freq. | % | Freq. | % | Freq. | % |
| Mombasa | 2,025 | 60 | 2.96 | 302 | 14.91 | 91 | 4.49 |
| Kwale | 3,536 | 107 | 3.03 | 627 | 17.73 | 175 | 4.95 |
| Kilifi | 3,585 | 93 | 2.59 | 587 | 16.37 | 172 | 4.80 |
| Tana River | 3,488 | 71 | 2.04 | 513 | 14.71 | 56 | 1.61 |
| Lamu | 2,792 | 74 | 2.65 | 372 | 13.32 | 74 | 2.65 |
| Taita/Taveta | 2,298 | 59 | 2.57 | 426 | 18.54 | 115 | 5.00 |
| Garissa | 3,119 | 29 | 0.93 | 188 | 6.03 | 36 | 1.15 |
| Wajir | 4,112 | 56 | 1.36 | 182 | 4.43 | 91 | 2.21 |
| Mandera | 4,453 | 252 | 5.66 | 731 | 16.42 | 266 | 5.97 |
| Marsabit | 3,210 | 144 | 4.49 | 407 | 12.68 | 78 | 2.43 |
| Isiolo | 3,161 | 78 | 2.47 | 195 | 6.17 | 163 | 5.16 |
| Meru | 2,884 | 80 | 2.77 | 441 | 15.29 | 120 | 4.16 |
| Tharaka-Nithi | 2,602 | 83 | 3.19 | 529 | 20.33 | 178 | 6.84 |
| Embu | 2,431 | 123 | 5.06 | 409 | 16.82 | 167 | 6.87 |
| Kitui | 2,728 | 67 | 2.46 | 473 | 17.34 | 120 | 4.40 |
| Machakos | 2,971 | 65 | 2.19 | 379 | 12.76 | 128 | 4.31 |
| Makueni | 3,031 | 61 | 2.01 | 436 | 14.38 | 109 | 3.60 |
| Nyandarua | 2,636 | 60 | 2.28 | 401 | 15.21 | 146 | 5.54 |
| Nyeri | 2,390 | 84 | 3.51 | 612 | 25.61 | 179 | 7.49 |
| Kirinyaga | 2,128 | 112 | 5.26 | 392 | 18.42 | 87 | 4.09 |
| Murang'a | 2,417 | 57 | 2.36 | 424 | 17.54 | 69 | 2.85 |
| Kiambu | 2,340 | 81 | 3.46 | 391 | 16.71 | 115 | 4.91 |
| Turkana | 3,578 | 111 | 3.10 | 671 | 18.75 | 313 | 8.75 |
| West Pokot | 4,097 | 94 | 2.29 | 285 | 6.96 | 85 | 2.07 |
| Samburu | 2,888 | 89 | 3.08 | 397 | 13.75 | 108 | 3.74 |
| Trans Nzoia | 3,218 | 75 | 2.33 | 306 | 9.51 | 73 | 2.27 |
| Uasin Gishu | 3,222 | 84 | 2.61 | 365 | 11.33 | 157 | 4.87 |
| Elgeyo/Marakwet | 3,271 | 68 | 2.08 | 291 | 8.9 | 123 | 3.76 |
| Nandi | 3,216 | 81 | 2.52 | 309 | 9.61 | 126 | 3.92 |
| Baringo | 2,604 | 114 | 4.38 | 339 | 13.02 | 111 | 4.26 |
| Laikipia | 2,650 | 77 | 2.91 | 323 | 12.19 | 87 | 3.28 |
| Nakuru | 2,698 | 38 | 1.41 | 257 | 9.53 | 85 | 3.15 |
| Narok | 2,921 | 115 | 3.94 | 380 | 13.01 | 116 | 3.97 |
| Kajiado | 2,325 | 64 | 2.75 | 226 | 9.72 | 125 | 5.38 |
| Kericho | 2,835 | 88 | 3.10 | 373 | 13.16 | 199 | 7.02 |
| Bomet | 3,428 | 71 | 2.07 | 395 | 11.52 | 121 | 3.53 |
| Kakamega | 3,470 | 168 | 4.84 | 610 | 17.58 | 130 | 3.75 |
| Vihiga | 3,192 | 131 | 4.10 | 439 | 13.75 | 123 | 3.85 |
| Bungoma | 3,654 | 120 | 3.28 | 393 | 10.76 | 86 | 2.35 |
| Busia | 3,575 | 197 | 5.51 | 713 | 19.94 | 236 | 6.60 |
| Siaya | 2,812 | 119 | 4.23 | 336 | 11.95 | 124 | 4.41 |
| Kisumu | 2,699 | 143 | 5.30 | 559 | 20.71 | 188 | 6.97 |
| Migori | 3,489 | 148 | 4.24 | 564 | 16.17 | 181 | 5.19 |
| Homa Bay | 3,176 | 174 | 5.48 | 465 | 14.64 | 88 | 2.77 |
| Kisii | 2,886 | 98 | 3.40 | 250 | 8.66 | 96 | 3.33 |
| Nyamira | 2,796 | 95 | 3.40 | 282 | 10.09 | 155 | 5.54 |
| Nairobi City | 1,998 | 80 | 4.00 | 267 | 13.36 | 135 | 6.76 |
| **Total** | **141,035** | **4,538** | **3.22** | **19,212** | **13.62** | **6,106** | **4.33** |
