# Supplementary material for: Socio-economic inequality and inequity in use of health care services in Kenya: evidence from the fourth Kenya household health expenditure and utilization survey
Source: Int J Equity Health. 2019 Dec 18;18:196. doi: 10.1186/s12939-019-1106-z (PMC6918604; doi:10.1186/s12939-019-1106-z)
Supplement: Supplementary file 2 — Additional file 2. Inequality and inequity in care use –comparison of ranking variables (total and non-food household expenditure). [file 12939_2019_1106_MOESM2_ESM.docx]

**Additional file 2 – Inequality and inequity in care use –comparison of ranking variables (total and non-food household expenditure)**

|  | Concentration index | | | | Horizontal inequity index | | | |
| --- | --- | --- | --- | --- | --- | --- | --- | --- |
| Care type | **Total hh. expenditure** | **Std. Err** | **Non-food expenditure** | **Std. Err** | **Total hh. expenditure** | **Std. Err** | **Non-food expenditure** | **Std. Err** |
| Outpatient | **0.0212** | *0.0040* | **0.0124** | *0.0039* | **0.0961** | *0.0040* | 0.0070 | *0.0039* |
| Preventive | **0.0256** | *0.0025* | **0.0201** | *0.0025* | **0.0156** | *0.0025* | **0.0132** | *0.0025* |
| Inpatient | **0.0149** | *0.0021* | **0.0128** | *0.0021* | **0.0048** | *0.0021* | **0.0065** | *0.0021* |
