# Supplementary material for: Socio-economic inequality and inequity in use of health care services in Kenya: evidence from the fourth Kenya household health expenditure and utilization survey
Source: Int J Equity Health. 2019 Dec 18;18:196. doi: 10.1186/s12939-019-1106-z (PMC6918604; doi:10.1186/s12939-019-1106-z)
Supplement: Supplementary file 3 — Additional file 3. Concentration curves for inpatient and outpatient care utilization by provider ownership. [file 12939_2019_1106_MOESM3_ESM.docx]

**Additional file 3 - Concentration curves for inpatient and outpatient care utilization by provider ownership**

| **Outpatient care** |
| --- |
| 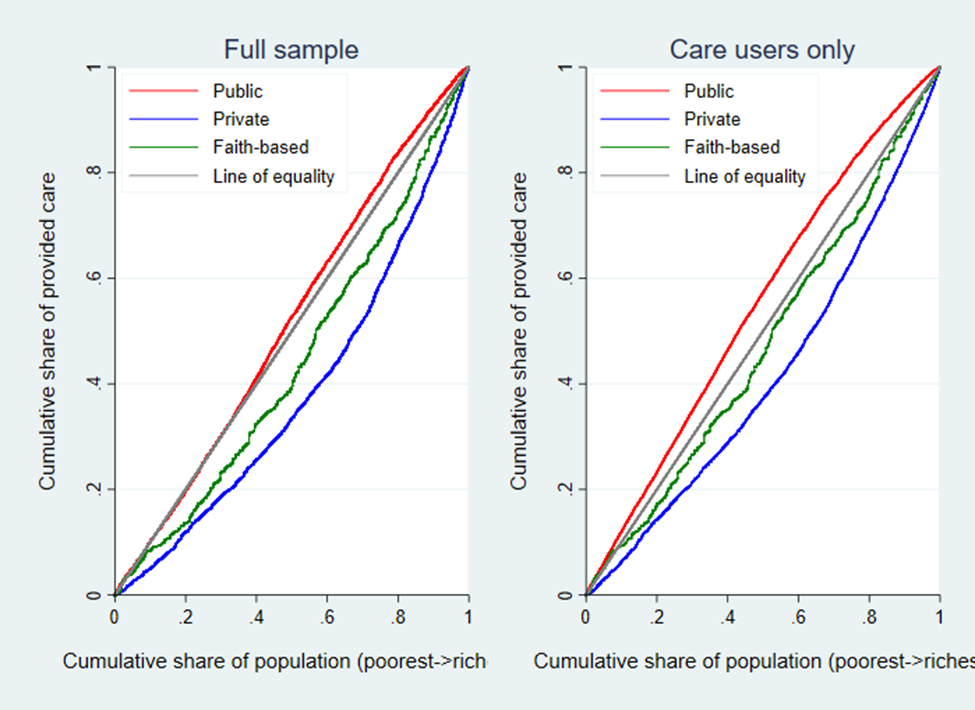 |
| **Inpatient care** |
| 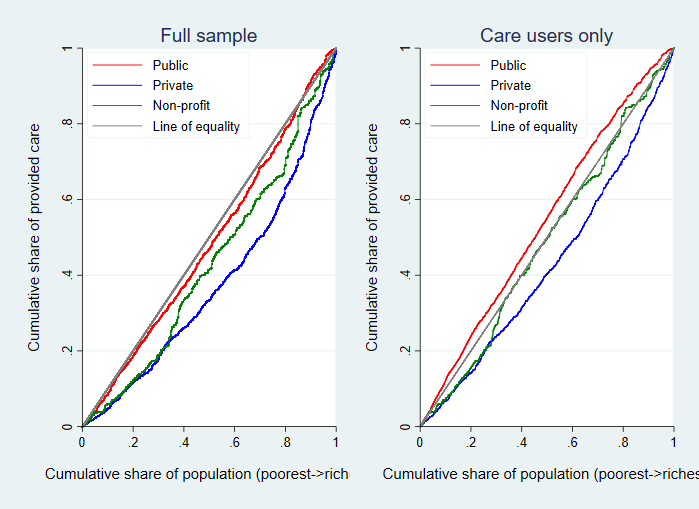 |
